# A facile synthesis of Ag_2_MnSnS_4_ nanorods through colloidal method

**DOI:** 10.55730/1300-0527.3435

**Published:** 2022-04-28

**Authors:** Sultan Süleyman ÖZEL, Serdar AKAY, Faruk ÖZEL

**Affiliations:** 1 Department of Materials Science and Engineering, Erciyes University, Kayseri, Turkey; 2 Department of Industrial Product Development, Production, and Design, Jönköping University, Jönköping, Sweden; 3 Department of Metallurgical and Materials Engineering, Karamanoğlu Mehmetbey University, Karaman, Turkey

**Keywords:** Ag_2_MnSnS_4_, AMTS, kesterite, nanorod, hot-injection

## Abstract

The production methods of semiconductor nanomaterials with new shapes and different compositions form the basis for the creation of high-performance structures in numerous applications. Kesterite structured materials are among these inorganic semiconductors and are suggested to be promising energy materials for the future. In this study, quaternary Ag_2_MnSnS_4_ nanocrystalline rods have been successfully synthesized for the first time by the colloidal hot-injection synthesis route and well-organized rod-like nanocrystals (NCs) with lengths ranging from 200 to 350 nm and widths from 10 to 30 nm were obtained. For this structure, the Ag_2_MnSnS_4_ exhibits a semiconductor property with a band-gap of approximately 1.3 eV. The optical properties and band-gap values were determined by UV-Vis absorption spectrum and using Tauc Equation. It has been observed that the Ag_2_MnSnS_4_ structure acquired by the proposed colloidal synthesis method can be an alternative to the commonly used materials based on Cd and Pb.

## 1. Introduction

Since the 20^th^ century, the demand for energy sources has continuously increased due to technological developments and increasing demand in society. Currently, many studies are focused on finding solutions to generate alternative energy sources to meet the increasing energy demand. These studies present new possibilities for current-generation applications in hydrogen harvesting and storage, lithium-ion batteries, supercapacitors, solar cells, photodiode, and carbon dioxide reduction processes [1–6]. Efficiency and production costs have been crucial factors for the usability of energy applications. Therefore, the materials that have been used in energy applications must be cost-effective and efficient, as well as non-toxic [1–8]. Recently colloidal nanomaterials have given rise to an exceptional class of emissive materials in sensing and bio-imaging, owing to optical, high luminescence properties, and excellent processability. However, as Cd and Pb-based chalcogenides are the precursor materials in this field and are toxic heavy metals, their industrial spread might pose a threat to human health, which has led to the search for different alternatives. To ensure compliance with existing safety regulations RoHS (Restriction of Hazardous Substances), there has been a crucial need for the development of non(less)toxic colloidal nanocrystals. Pb/Cd-free, ((Ag,Cu)_2_XSn(S,Se)_4_ X:Zn, Mn, Ni, Co) NCs produced by the hot injection synthesis route are non(less) toxic materials [9]. As an alternative to Cd and Pb-based chalcogenide materials, NIR-emitting indium/gallium-based group nanomaterials such as CuInGaS (CIGS), InSb, GaAs_7,_ and CuIn (S/Se)_2_, AgIn (Se/Te) have received attention. The need for alternative materials has continued to be examined because the lack of indium/gallium in the Earth’s crust could limit large-scale commercial agreements. Recent studies showed that quaternary semiconductors of ((Ag, Cu)_2_XSn (S,Se)_4_) with a small band-gap, have shown NIR luminescence and are considered to be a potential alternative in this field of research [10,11]. The most interesting of these materials is Cu_2_ZnSnS_4_ (CZTS) NCs and it is shown as one of the energy materials of the future with its superior properties. CZTS NCs, which exhibit semiconductor properties, have direct band-gap properties and high absorption coefficients in the visible region (~10^4^ cm^−1^). The band-gap of CZTS NCs has about 1.6–1.4 eV, which is close to the optimum value for solar cell application [12]. Compared to the CIGS structure, CZTS is non(less)toxic and the precursor elements are abundant on the earth (Zn and Sn are 1500 times and 45 times greater than that of In, respectively).

Recently, Ag_2_XSnS_4_ NCs, which have similar properties to CZTS NCs, have taken their place among the new remarkable structures in this field. In the literature review, only a few studies were found on the synthesis of Ag_2_XSnS_4_ NCs, and the details of these studies are given in the following lines. According to the study by Saha et al. they synthesized Ag_2_ZnSnS_4_ (AZTS) NCs by the colloidal synthesis technique. Their NCs were in a spherical shape with a 6 nm size and 1.5 eV band-gap [9]. In another report, Pietak et al. prepared high-quality AZTS NCs of various sizes with average band-gap energy of 2.0 eV by the chemical vapor transport (CVT) method [13]. Additionally, Kumar et al. reported that they produced the (AgxCu_1-x_)_2_ZnSnS_4_ (ACZTS) structure using the thin film method and ACZTS structure has different ratios (x:0–0.04) 1.6 eV, 2.4 eV band-gap respectively [14]. Amongst them, AMTS is one of the structures of recent interest as it demonstrates antiferromagnetic behaviors. However, an important limitation of the studies on AMTS mentioned is that solid-state and melt-annealing techniques requiring high temperature and complex processes are used for the synthesis of these structures [15,16].

In this study, AMTS nanorods (NRs) were obtained for the first time by the colloidal synthesis route, which is considered a more economical, Pb/Cd-free, and versatile route than previous techniques. Therefore, it is suitable for additive fabrication techniques such as printing and direct writing for large-scale or specific applications. The band-gap of obtained NRs by the proposed synthesis technique were calculated 1.3 eV. This indicates that AMTS NRs can be a promising absorbent material for energy conversion applications.

## 2. Experimental section

### 2.1. Materials

To perform experimental process, silver nitrate (AgNO3 99.99%), sulfur powder (99.5%), tin (II) acetate (Sn(OAc)2–99.99%), propanol (%99.5), ethanol (%99.8) were obtained from Sigma-Aldrich. Manganese (II) acetate (Mn(OAc)2-(%95) was purchased from Alfa-Aesar. Oleylamine (OAm %98.5), 1-Dodecantiol (DDT-%98), Ter-Dodecylmercatan (t-DDT) were purchased from Acros Organics. Toluene was provided by VWR.

### 2.2. Colloidal synthesis of AMTS NRs

**Figure 1:**
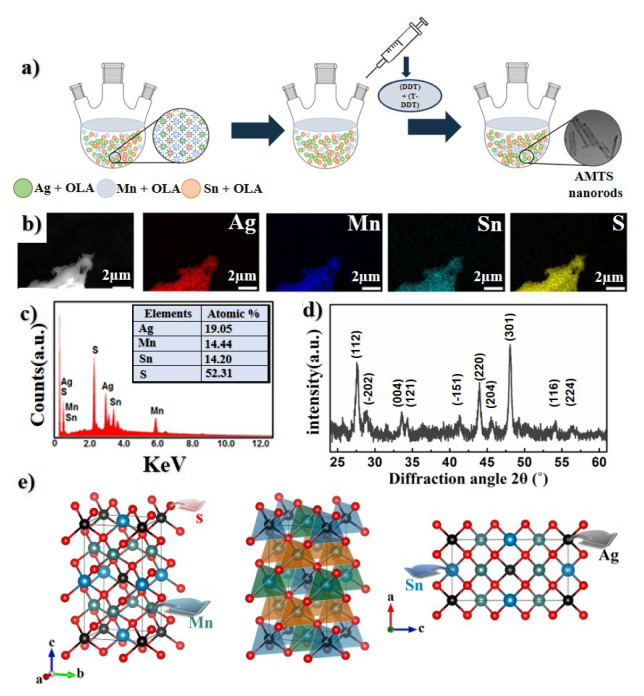
Schematic illustration of the colloidal synthesis of the AMTS NRs (a), EDS elemental mapping images (b), EDS spectrum (c) XRD pattern (d), and 3D-crystal structure (e) of the AMTS NRs.

AMTS NRs were synthesized by previously published CZTS nanocrystal synthesis methods with a small extent modification [10,17]. A schematic illustration of the production process is given in Figure 1a. Briefly, 0.5 mmol AgNO_3_, 0.25 mmol Mn(OAc)_2_, 0.25 mmol Sn(OAc)_2_, and 15 mL OAm were added into a 50 mL three-neck flask and mixed under Ar gas flow, allowing the precursors to dissolve and the reaction medium to be deoxygenated. The reaction medium was heated until the solution temperature reached 100 °C, and at this temperature, 0.13 mL of DDT & 0.88 mL of the t-DDT mixture were injected into the solution. Afterward, the solution was instantly heated up to 240 °C and kept by stirring at this temperature for 30 min to complete the formation of the NRs. Fourteen mL of toluene and two mL of propanol were added to the obtained solution for particle formation and it was precipitated by centrifuging at 3000 rpm for 1 min. Finally, the resulting precipitate was washed with ethanol and left to dry.

### 2.3. Characterization

The surface morphology of as-prepared AMTS NRs was acquired by using the scanning electron microscope (SEM-Hitachi SU5000, Japan). The size distribution of the NRs and statistical data were analyzed by using the data program (Origin). The structure was analyzed via an X-ray diffractometer (Bruker D8, Germany) (CuKα source, λ = 1.5406 nm). Fast mapping and composition of the NRs have obtained SEM–EDS instruments (SEM–EDS Hitachi SU5000, Japan). The composition of AMTS NRs was analyzed by using an inductively coupled plasma-mass spectrometer (ICP-MS AGILENT 7500A, The United States). A confocal Raman microscope (Alpha 300 M+, WITec, Germany) was used for Raman spectrometry measurements. The morphology, crystalline structure, and size distribution were investigated using a transmission electron microscope (TEM) (JEOL JEM-2100F, Japan). The absorbance spectrum and optic band-gap of AMTS NRs were acquired and calculated by using a Shimadzu UV-3600 UV–vis–NIR spectrometer (Shimadzu UV-3600, Japan).

## 3. Result and discussions

The synthesis process of AMTS NRs was carried out in sequential and different processes. In the first step, the solution is formed by dissolving the precursor materials (Ag, Mn, Sn) in OLA. In the second step, the Ag_2_S nucleus begins to form following the addition of the sulfur sources (DDT and t-DDT) in the synthesis medium (Figure 1a). Finally, by increasing the temperature, Mn and Sn atoms started to diffuse through the Ag_2_S nucleus and the final rod particles were obtained by growing the nucleus with different chain structures of sulfur sources in the environment [18–21].

Figure 1b shows the EDS maps for the AMTS NRs. The mapping results bring to light that the elements in the AMTS structure are homogeneously distributed. According to the results of the EDS analysis, the atomic ratio of Ag, Mn, Sn, and S for the AMTS sample is estimated to be 0.38, 0.29, 0.28, and 1.05, respectively. These values are reasonably close to the ratios of 0.5, 0.25, 0.25, and 1 that are expected for the AMTS phase structure. The mean elemental composition (%) ratios of the NRs, given in Figure 1c, are close to the theoretical values. Also, the average composition of AMTS NRs was confirmed by ICP-MS as shown in Table. When the results are analyzed, it is clearly seen that the obtained values are quite close to the targeted composition value. In addition, the obtained results are compatible with the EDS results, confirming that the synthesized NRs have been successfully obtained.

**Figure 2:**
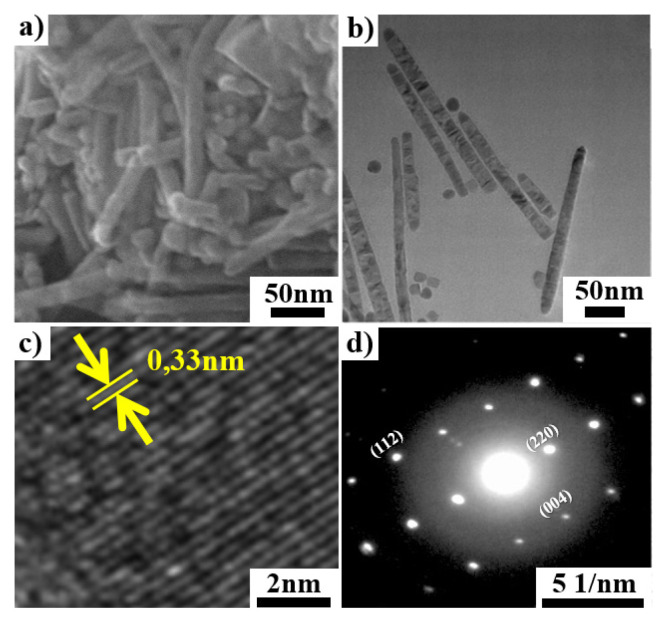
SEM image (a), TEM image (b), HR-TEM image (c), and SAED pattern (d) of AMTS.

It can be concluded from these results that it has been possible to obtain silver-based kesterite particles with nanorod structure by using the colloidal hot-injection method. Figure 1d shows the XRD pattern of the obtained NRs. The main diffraction peaks manifested themselves at about 2θ of 27.6°, 33.6°, 34.3°, 44.0°, 45.5°, 48.08°, 54.1°, and 56.4° which were assigned to (112), (004), (121), (220), (204), (301), (116), and (224) planes, respectively. The observed peak values agree with the tetragonal pirquitasite (JCPDS 35–435) structure. The unit cell of this structure consists of 23 polyhedra centered on Ag, Mn, and Sn atoms, and in this structure, each cation atom bonds with 6 sulfur atoms (Figure 1e). These results are consistent with previous reports conducted on AZTS and CZTS structures [9,22]. Moreover, diffraction peaks coming from the secondary Ag_2_SnS_3_ phase at 2θ: 28.9° and 41.5° can be identified and their attributes to (−202) and (−151) planes, respectively. The average crystallite size of obtained AMTS NCs has been calculated to be 23.3 nm using the Scherrer equation which is consistent with the particle widths observed by SEM (Figure 2a) and TEM (Figure 2b). Figure 2a and b present the typical SEM and TEM images of as-synthesized NRs. As shown in Figure 2 (a and b), the synthesized particles have mostly rod-shaped structures. It has been determined that the width of the particles varies between 10 to 30 nm, and the lengths vary between 200 to 350 nm. Figure 2 (c) shows a high-resolution TEM (HR-TEM) image of the NRs. The lattice fringe observed by the HR-TEM image is 0.33 nm, which corresponds to the (112) lattice plane of body-centered tetragonal AMTS (Figure 2c). Moreover, selected area electron diffraction (SAED) patterns of the NRs have been given in Figure 2d and indexed (112), (220), and (004) planes confirmed that the synthesized particles were in tetragonal-type of AMTS. The dotted structure is seen in the SAED pattern, which indicates a high degree of crystallinity and also confirms that the nature of the particles is single crystal. These results match the XRD and HR-TEM results.

**Figure 3:**
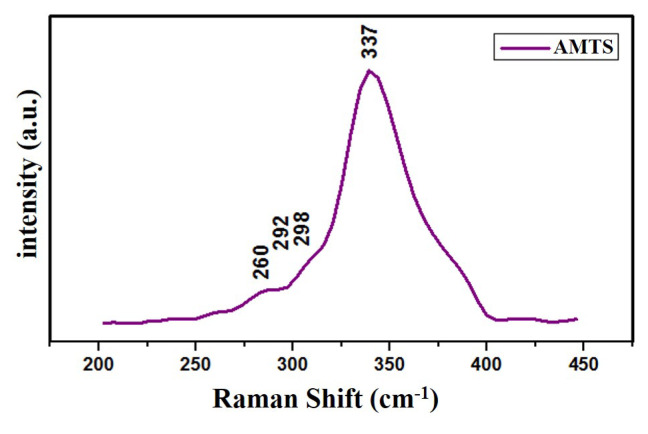
Raman spectrum of the AMTS NRs.

Raman analysis was performed to determine the additional phase states that cannot be seen in XRD analysis. The Raman spectrum of the NRs (λexc = 514.7 nm) is shown in Figure 3. The spectrum exhibits a major peak at 337 cm^−1^ approximately, which is generally donated as the A_1_ vibration mode which originates from the vibration of the sulfur sublattice [23,24]. Moreover, the NRs also exhibit peaks such as AMTS (337 cm^−1^) [25], MnS (292 cm^−1^) [26], and Ag_2_SnS_3_ (260 and 298 cm^−1^) [27], all of which can be attributed to AMTS and all these peaks are in line with the reported results. Furthermore, the absence of any additional peaks from possible secondary phases also confirmed the purity of the synthesized NRs.

**Figure 4:**
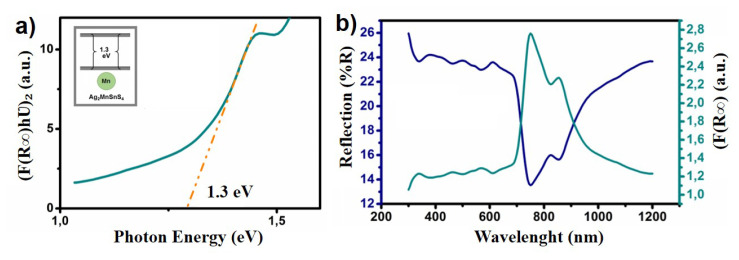
UV-vis and band-gap energy diagrams of the AMTS NRs (the inset shows the schematic illustration of calculated band-gap structure) (a) and diffuse reflection and absorption spectra (b).

The optical properties, band-gap values, and species of the NRs were determined by using UV–vis absorption spectrum and diffuse reflection spectra. UV–vis absorption spectrum, diffuse reflection, and absorption spectra of the AMTS NRs are shown in Figure 4 (a, b). The optical band-gaps of these NRs were measured at around 1.3 eV by using Tauc Equation [28,29]. The obtained band-gap value is close to the optimal values reported in the literature for solar cell applications. For example; Li et al. have calculated Cu_2_ZnSn(S1−_x_Sex)_4_ NCs changing from 1.5 eV to 1.12 eV band-gaps [30], on the other hand, Su et al. have found CZTS nanowires and nanotubes 1.57 eV, 1.61 eV band-gaps respectively [31]. Also, Yıldırım et al. have determined 1.51 eV which is similar to the earlier report [32].

## 4. Conclusion

In summary, colloidal AMTS NRs were successfully synthesized using a low-cost and facile colloidal hot-injection method. XRD, Raman, ICP-MS, and EDS measurements confirmed the stoichiometry of the AMTS structure. The morphology of the NRs was determined by SEM and TEM analysis, where the results show that the width varies between 10 to 30 nm, and the lengths vary between 200 to 350 nm. Moreover, HR-TEM and SAED images showed that the NRs are single crystalline. In conclusion, with this work, we demonstrate the first example of AMTS NRs by expanding the material availability of Pb/Cd-free colloidal nanoparticles.

## Figures and Tables

**Table:** Composition of AMTS NRs measured ICP-MS.

Ag(%)	Mn(%)	Sn(%)	S(%)	Ag/(Mn+Sn)	Mn/Sn
22.4	13.2	12.9	51.5	~ 0.86	~ 1.0
